# Phthalic Acid Chemical Probes Synthesized for Protein-Protein Interaction Analysis

**DOI:** 10.3390/ijms140712914

**Published:** 2013-06-24

**Authors:** Shih-Shin Liang, Wei-Ting Liao, Chao-Jen Kuo, Chi-Hsien Chou, Chin-Jen Wu, Hui-Min Wang

**Affiliations:** 1 Department of Biotechnology, College of Life Science, Kaohsiung Medical University, 100, Shih-Chuan 1st Road, San-Ming District, Kaohsiung 807, Taiwan; E-Mail: wtliao@kmu.edu.tw; 2 Center for Resources, Research and Development, Kaohsiung Medical University, 100, Shih-Chuan 1st Road, San-Ming District, Kaohsiung 807, Taiwan; E-Mail: chishien@kmu.edu.tw; 3 Graduate Institute of Medicine, College of Medicine, Kaohsiung Medical University, 100, Shih-Chuan 1st Road, San-Ming District, Kaohsiung 807, Taiwan; E-Mail: achilleskuo@ntu.edu.tw; 4 Kaiser Pharmaceutical Co., LTD., 9, Huangong Road, Yong-Kang District, Tainan 710, Taiwan; E-Mail: ken@kaiser.com.tw; 5 Department of Fragrance and Cosmetic Science, College of Pharmacy, Kaohsiung Medical University, 100, Shih-Chuan 1st Road, San-Ming District, Kaohsiung 807, Taiwan; E-Mail: davidw@kmu.edu.tw

**Keywords:** phthalate, phthalic acid, chemical probe, protein-protein interaction

## Abstract

Plasticizers are additives that are used to increase the flexibility of plastic during manufacturing. However, in injection molding processes, plasticizers cannot be generated with monomers because they can peel off from the plastics into the surrounding environment, water, or food, or become attached to skin. Among the various plasticizers that are used, 1,2-benzenedicarboxylic acid (phthalic acid) is a typical precursor to generate phthalates. In addition, phthalic acid is a metabolite of diethylhexyl phthalate (DEHP). According to Gene_Ontology gene/protein database, phthalates can cause genital diseases, cardiotoxicity, hepatotoxicity, nephrotoxicity, *etc.* In this study, a silanized linker (3-aminopropyl triethoxyslane, APTES) was deposited on silicon dioxides (SiO_2_) particles and phthalate chemical probes were manufactured from phthalic acid and APTES–SiO_2_. These probes could be used for detecting proteins that targeted phthalic acid and for protein-protein interactions. The phthalic acid chemical probes we produced were incubated with epithelioid cell lysates of normal rat kidney (NRK-52E cells) to detect the interactions between phthalic acid and NRK-52E extracted proteins. These chemical probes interacted with a number of chaperones such as protein disulfide-isomerase A6, heat shock proteins, and Serpin H1. Ingenuity Pathways Analysis (IPA) software showed that these chemical probes were a practical technique for protein-protein interaction analysis.

## 1. Introduction

Phthalic acid is a precursor utilized in the manufacture of phthalates, which are extensively used in the production of plastic goods to increase their flexibility and for ease of softening. Phthalic acid is a hydrocarbon plasticizer that is converted to phthalates by esterification at 2 carboxyl functional groups. Humans are invariably exposed to plasticizers as adducts in Saran wrap, toys, cosmetics, medical tubes, blood bags, plastic bags, and plastic bottles. Patients undergoing hemodialysis are also exposed to diethylhexyl phthalate (DEHP) and polyvinyl chloride (PVC) [1,2]. According to a report by the U.S. Centers for Disease Control and Prevention (CDC), over 97% Americans were found to have phthalate metabolites suchas monoethyl phthalate (MEP), monobutyl phthalate (MBP), and monobenzyl phthalate (MBzP) in their urine, and over 75% Americans were found to have mono-2-ethylhexylphthalate (MEHP) and monomethyl phthalate (MMP) in their urine [3,4]. Urine samples of over 92% Americans were also contaminated with bisphenol A (BPA) [4,5].

In previous reports, phthalates have been shown to play an essential role in causing asthma [6] and in advancing breast cancer progression by promoting the overexpression of lymphoid enhancer factor 1 [7]. Moreover, phthalates are detected in cosmetic products [8] and phthalate metabolites are classified as teratogenic or endocrine disruptors [8–10]. Further, simulation results from the Comparative Toxicogenomics Database (CTD) have shown that phthalates are associated with cardiotoxicity, hepatotoxicity, genital diseases, and nephrotoxicity [11].

In recent years, proteomics research has emphasized on disease target protein mining coupled with biomarker evaluation [12] and verification [13]. However, discovery of target proteins and protein-protein interaction need to be efficiently discovered via analytic methods coupled with mass spectrometry (MS). Currently, comparisons between normal and abnormal specimens by genomics and quantitative proteomic techniques such as multi-dimensional gel analyses [14,15] or gel-free shotgun proteomics coupled with liquid chromatography (LC) tandem MS [16] to discover novel protein markers for early cancer detection [17] or mRNAs as biomarkers are particularly attractive. At the verification and validation stages, microarray chips [18,19], ELISA chips [20,21], qPCR [22], and triple quadrupole MS using the multiple reaction monitoring scanning mode (MRM) [23–25] are widely accepted approaches. However, biomarker selection is difficult because thousands of candidates of proteins can be identified using powerful MS and improved separation techniques.

In this study, we focused on protein-protein interaction having the ability to bind phthalate by generating chemical probes using phthalic acid with ATPES linker on a carrier. Designing of endocrine disruptor molecules on carriers to demonstrate protein-protein interaction is a novel concept. According to a previous study, phthalic acid, being a superior metabolite of DEHP, was detected in urine, serum, and dialysate samples [2]. Phthalic acid was detected in urine because it is delivered from the kidneys through the ureter to the bladder. Thus, we used a normal rat kidney cell line (NRK-52E) to examine protein-protein interactions between phthalic acid probes and NRK-52E cell lysates. In addition, when designing chemical probes to investigate proteomes, it was shown that protein profiling by activity-based chemical probes [26] and activity-based proteomics were linked with enzymes such as serine hydrolases [27]. Therefore, it was possible to design probes by covalent bonding between proteins and carriers using click chemistry [26,28], nano-gold DNA probes synthesized for MS detection [29], and chemiluminescent bioprobes [30]. In previous studies, the metabolite phthalic acid derived from phthalates were observed in dialysis patients [2]. Phthalic acid metabolized from DEHP and MEHP [31] was also demonstrated. Further, chemical probes can be modified and applied to investigate the feasibility of direct binding and protein–protein interactions such as utilization of affinity tags to purify proteins [32].

Here, composites of phthalic acid chemical probes were generated using phthalic acid activated by 1-ethyl-3-(3-dimethylaminopropyl) carbodiimide/*N*-Hydroxysuccinimide (EDC/NHS) and silanized APTES–SiO_2_. The chemical probes were utilized for protein-protein interactions as well as coupled with phthalic acid. Thus, a statistical list of proteins that were bound by phthalic acid was generated on the basis of high ratios of isotope labeling. In protein search and network establishing, Mascot Distiller [33] was utilized for protein identification and quantitation, and Ingenuity Pathways Analysis (IPA) [34] was used for protein-protein interaction.

## 2. Results and Discussion

### 2.1. Procedures for Chemical Probe Synthesis

**Figure 1:**
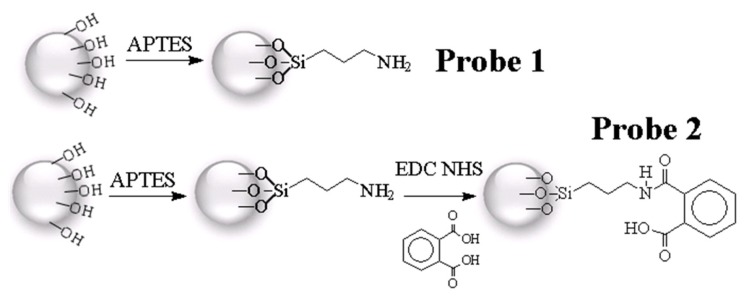
Schematic procedure for generation of chemical probes.

Figure 1 shows the scheme by which SiO_2_ particles were activated using 0.5 M NaOH and 0.5 M HCl, washed with deionized water, and dried with ethanol. The surface of SiO_2_ modified by APTES through silanization displayed an amino group as the functional reactive site [35]. In addition, the carboxylic group of phthalic acid was activated by EDC/NHS [36]. After 12 h of silanization, the amino group on APTES-modified SiO_2_ reacted with phthalic acid to generate amide bonds.

### 2.2. Characterization of Chemical Probes by IR Spectrometry

**Figure 2:**
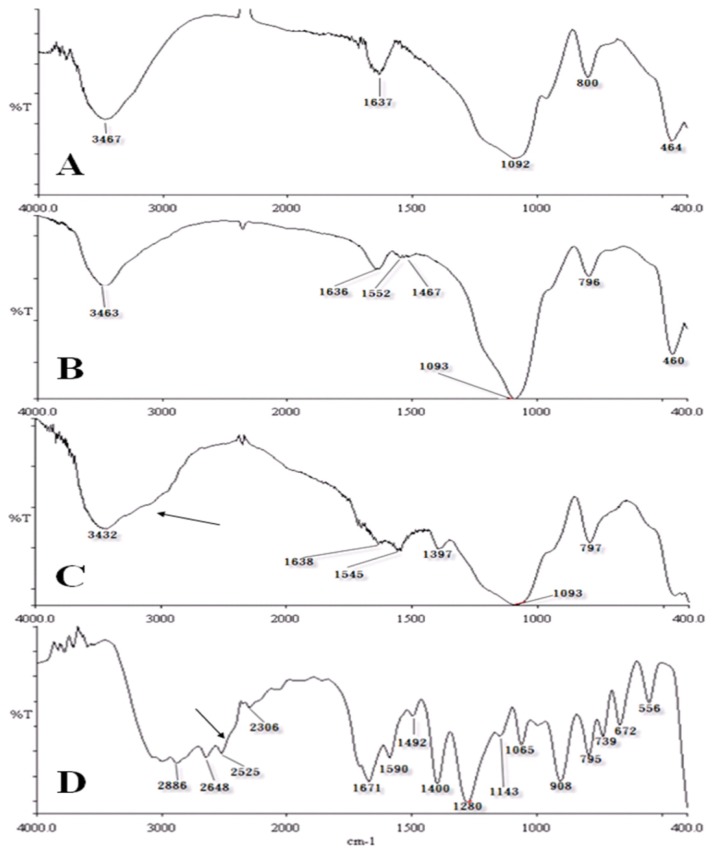
Infrared (IR) spectra of (**A**) 400 mesh SiO_2_ particles; (**B**) Silanized SiO_2_ with 3-aminopropyl triethoxyslane (APTES); (**C**) Phthalic acid activated by 1-ethyl-3-(3-dimethylaminopropyl) carbodiimide/*N*-Hydroxysuccinimide (EDC/NHS) and linked with silanized SiO_2_; (**D**) Pure phthalic acid powder.

Probes 1 (APTES-modified chemical probes) and Probe 2 (phthalic acid probes with an APTES linker) were characterized using IR spectroscopy. In Figure 2A,B, the spectrum of APTES–SiO_2_ showed peaks at wave numbers 1552 cm^−1^ and 1467 cm^−1^ which was different from that of SiO_2_. The IR spectra of chemical probes with SiO_2_ modified by APTES and phthalic acid are shown in Figure 2C and that for pure phthalic acid is shown in Figure 2D. Black arrows indicate a similar spectrum from 3000 to 2300 cm^−1^ in Figure 2D; the peak for phthalic acid shifted from 1400 to 1397 cm^−1^. Figure 2C shows a weak peak near 1368 cm^−1^; this peak was similar to that for phthalic acid at 1671 cm^−1^ in Figure 2D.

### 2.3. LC-MS/MS for Identification and Quantitation of Bound Proteins

**Figure 3:**
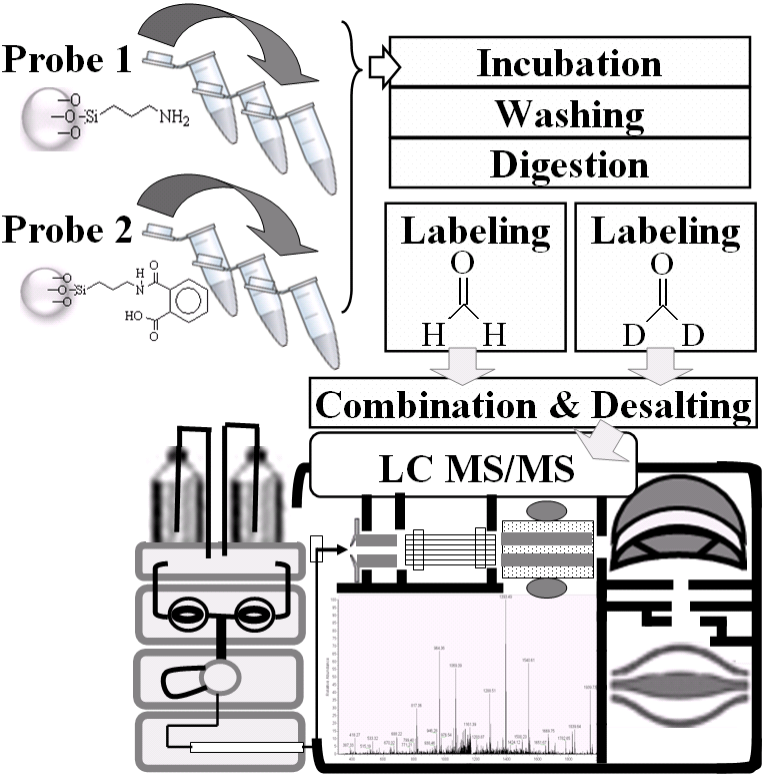
Flow chart and sample pretreatment for quantitative approaches in proteome analyses coupled with nano-liquid chromatography (LC)-mass spectrometry (MS)/MS for protein identification and quantification.

Chemical probes were generated in different manners either with APTES (Probe 1 in Figure 1) or EDC/NHS conjugated with APTES and phthalic acid (Probe 2 in Figure 1) as shown in the flowchart in Figure 3. These chemical probes were incubated with NRK-52E cell lysates, diluted, incubated at 37 °C and then washed. Following reduction, alkylation, and tryptic digestion, Probe 1 and Probe 2 were labeled with formaldehyde-*H*_2_ and formaldehyde-*D*_2_, respectively. Samples of Probe 1 and Probe 2 were combined in an Eppendorf tube and applied to a C18 desalting column prepared in-house.

**Figure 4:**
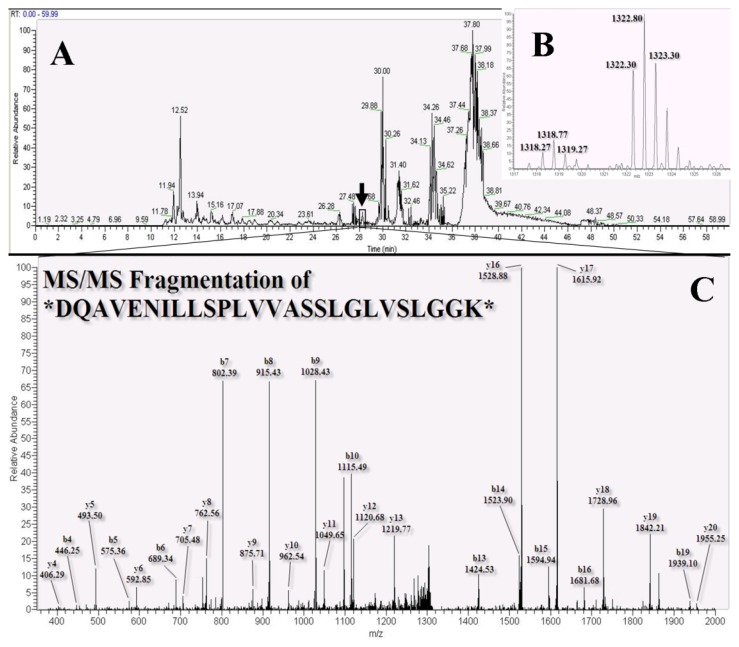
Nano-LC-MS/MS analytic spectra for tryptic digestion of epithelioid cell lysates of normal rat kidney (NRK-52E) cell lysates generated with disparate phthalic acid chemical probes (**A**) Base peak chromatograph of the NRK-52E binding peptides; (**B**) Mass spectrometric determination for summing 3 isotope peak areas of a peptide that was labeled with light (L, formaldehyde-*H*_2_) and heavy (H, formaldehyde-*D*_2_) isotopes showing the ratio of the DQAVENILLSPLVVASSLGLVSLGGK peptide with a 2^+^ charge; (**C**) Product ion scan spectrum of the peptide DQAVENILLSPLVVASSLGLVSLGGK (*m/z* 2642.58) that is found in serpin H1 protein. Asterisks indicate the amino acids generated by dimethyl labeling reagents, and the y-ions (*C*-terminal fragments) as well as b-ions (*N*-terminalfragments) are produced by fragmentation by the collision gas.

As shown in Figure 3, we developed these samples using nano-LC-MS/MS and identified disparate proteomes using different chemical probes and quantitative reagents. We determined that 60 min of an elution gradient was required for separation (Figure 4A) and that the chromatography of peptides labeled with different isotopes had the same chemical qualities. These could be co-eluted and detected by MS. Using dimethyl labeling technique [37], proteins bound to Probe 1and Probe 2 could be distinguished by the variation in the molecular weights of formaldehyde-*H_2_* and formaldehyde-*D*_2_ (4 Da and 8 Da, respectively). The ratios of hydrogen-labeled peptides against deuterium-labeled peptides (heavy/light; D/H) were analyzed by the Mascot Distiller software using the average areas of the first three isotopic peaks across the elution profile. This showed disparate peptide quantities (Figure 4B). Protein quantitation and protein identification were also performed using the software. In the protein identification program, the highest signal intensities of five peptides carrying charges 2^+^ and 3^+^ were selected in the collision chamber to be mixed with nitrogen gas and to be broken at the peptide bond to form the b-ions and y-ions (Figure 4C). In the MS spectrum, the b-ions and y-ions were labeled to show integration of the peptide sequence of Serpin H1 (black arrow in Figure 4A, peptide sequence: DQAVENILLSPLVVASSLGLVSLGGK, 2^+^, retention time 28.3 min) protein. Stable isotope dimethyl labeling, occurring on the *N*-terminal and amino group of lysine (K), is indicated with an asterisk and invariably resulted in a mass shift (four methyl groups on the *N* terminal and lysine) from *m/z* 2578.46 to *m/z* 2634.52 (formaldehyde-*H_2_*) and *m/z* 2642.58 (formaldehyde-*D_2_*). All of the protein identification and quantitation raw data and integrated statistics reports are given in electronic supplementary information (ESI).

### 2.4. Functions of Phthalate Chemical Probe Binding Proteins

**Figure 5:**
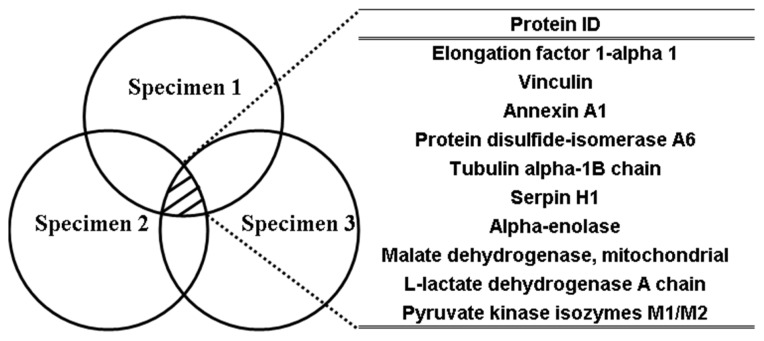
Triplicate experiments from chemical probes generated with NRK-52E lysates, intersection of triplicate experiments developed a protein list in which proteins score above 90, and at least the ratio (D/H) >3 of two experiments.

Proteins were identified by peak lists that were converted from the nano-LC-MS/MS spectra using the Mascot searching engine against mouse taxonomy in the Swiss-Prot database for exact matches using the Mascot Distiller software. In Figure 5, proteins identified by nano-LC-MS/MS in triplicate experiments are shown with black shades overlapped within the three open circles and listed in the corresponding table. The raw data are given in ESI. The criteria used for protein selection were protein list score >80 and a D/H ratio of at least >3 in replicate experiments.

Based on these criteria, the identified proteins included elongation factor 1-alpha 1, vinculin, annexin A1, protein disulfide isomerase A6, tubulin alpha-1B chain, serpin H1, alpha-enolase, malate dehydrogenase, l-lactate dehydrogenase A chain, and pyruvate kinase isozymes M1/M2. These are listed in Figure 5.

**Figure 6:**
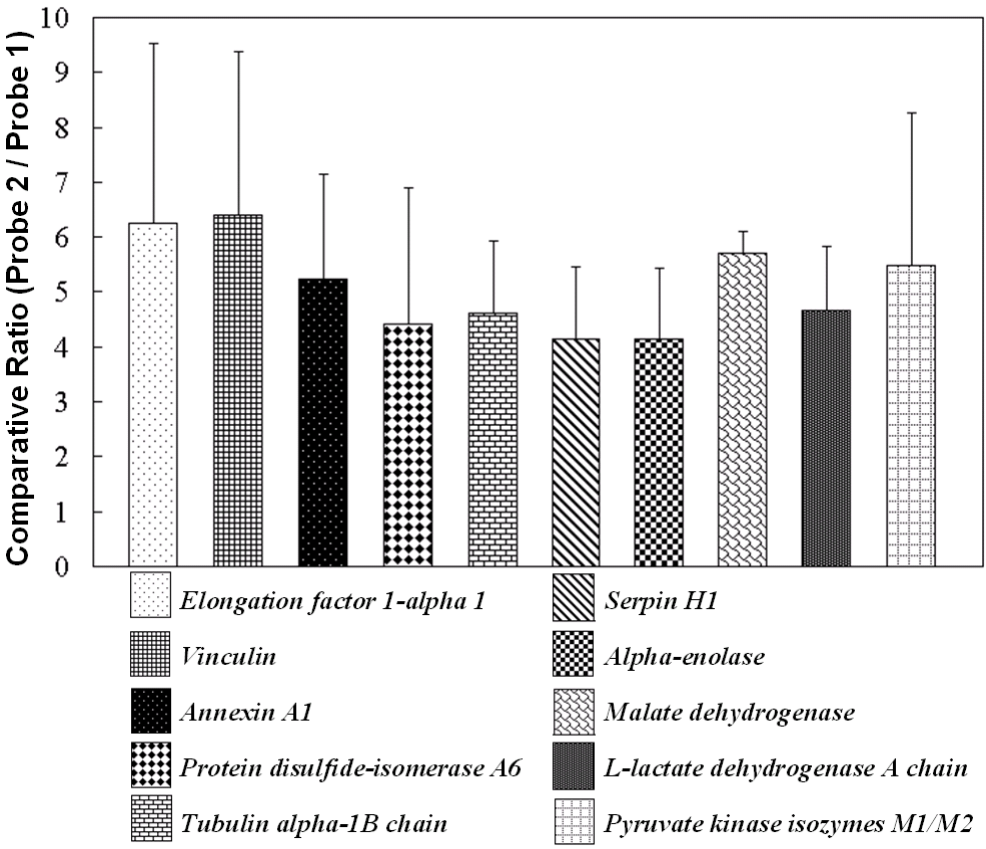
Comparative ratio of Probe 1 and Probe 2 that interacted with NRK-52E cell lysate proteins in three experiments.

Phthalate chemical probes were used to narrow down the target protein or protein-protein interaction candidates determined by LC-MS/MS. Based on the statistics data for the protein list (Figure 5), there were two proteins involved in chaperone functions; one was protein disulfide isomerase A6 that affects the aggregation of platelet, and the other was serpin H1 that is involved in the collagen biosynthesis pathway. In addition, elongation factor 1-alpha 1 with a D/H average ratio of 6.26 plays an important role in protein synthesis. Regarding energy generation, malate dehydrogenase, a mitochondria-related proteins, reversibly catalyzes the formation of NAD^+^ from NADH. l-lactate dehydrogenase A chain and glyceraldehyde-3-phosphate dehydrogenase also exhibit the same reaction. NADH can be utilized in the electron transport chain (ETC) to generate ATP via a proton gradient and by electron donation. Phthalic acid probes also bound to ATP-generating pyruvate kinase isozymes M1/M2. Alpha-enolase exerts its activity prior to the catalysis of pyruvate kinase in glycolysis. Furthermore, ATP synthase subunits alpha and beta are crucial components involved in ATP synthesis. However, these were not listed in the table because of insufficient scores or ratios. Annexin A1 is a calcium/phospholipid-binding protein. In a previous report, butyl benzyl phthalate (BBP) blocked calcium signal transduction by stimulation of purinoceptors [38]. From the raw data of triplicate experiments, many proteins were identified on the basis of D/H ratios below 1. These results demonstrated that non-specific binding could be eliminated by using two types of chemical probes. However, proteins with non-specific binding were absorbed on Probe 1 and Probe 2 because of the same APTES linker. The statistics data in Figure 6, presents the proteins with D/H ratios of >3-fold proteins were presented at three times experiments.

**Figure 7:**
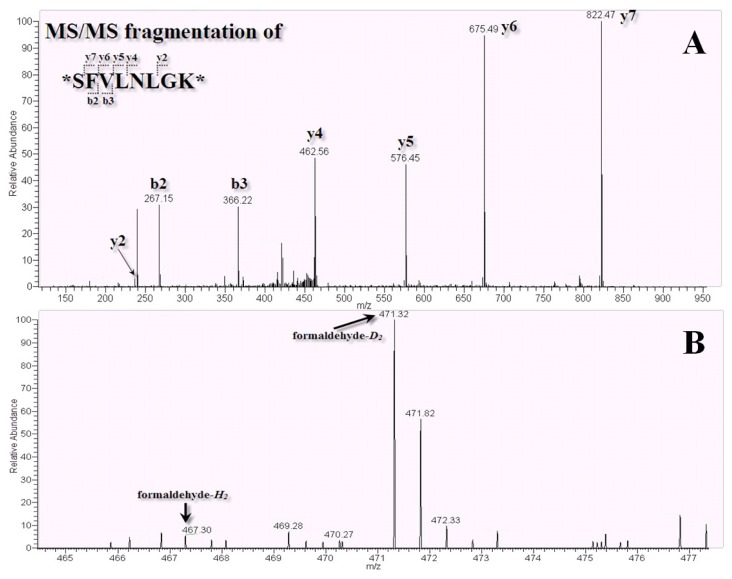
(**A**) Protein identification of galectin-1 using the MS/MS spectrum of the amino acid sequence SFVLNLGK (*m/z* 471.32; 2^+^) was determined with the fragmented y-ion and b-ion; (**B**) Peptide SFVLNLGK of galectin-1 showed the ratio offormaldehyde-*H_2_* labeled peptide (*m*/*z* 467.30) to formaldehyde-*D_2_* labeled peptide (*m*/*z* 471.32).

The protein galectin-1 shown in Table 1 had significantly increasing ratios of 40-, 9-, and 14-fold, and in experiment 2, galectin-3 had a 12-fold increase. It has been reported that galectin-1 is involved in multifarious functions such as cell migration, cell adhesion, cell proliferation, inflammation and apoptosis [39]. As shown in Figure 7, the peptide SFVLNLGK belonging to galectin-1 was observed in the LC-MS/MS (fragmentation of peptide shown in Figure 7A), and the ratio difference for galectin-1 was obvious. In Figure 7B, *m/z* 467.3 corresponded to labeled formaldehyde-*H*_2_ and *m/z* 471.32 corresponded to labeled formaldehyde-*D*_2_. However, from the raw statistics data, the ratios of each protein in triplicate experiments showed the same increasing trends, although the variations in these values were considerable. The NRK-52E cell lysates and chemical probes were incubated in an equilibrated environment. Therefore, Probe 2 showed a priority to bind with those proteins that had higher affinities as well as those that were bound to Probe 1 in low abundance. Thus, the phthalic acid Probe 2 design had the potential to discover and monitor protein-protein interaction.

### 2.5. IPA: Pathways for Protein-Protein Interactions

**Figure 8:**
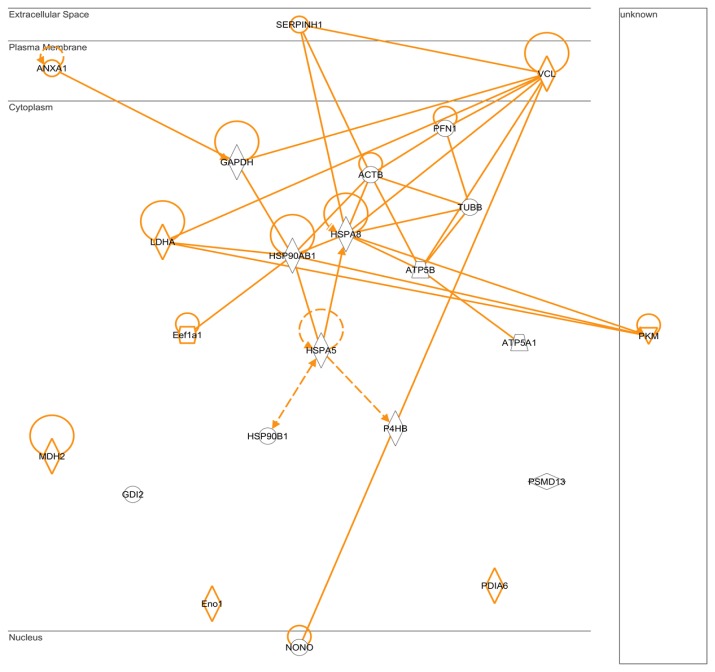
Schematic representation of the pathways and networks of 20 proteins, which function as chaperones and are associated with ATP generation, with high affinity ratios for phthalic acid chemical probes.

The proteins interacting with a chemical probe were analyzed using IPA software by selecting protein-protein interactions. As shown in Figure 8, proteins such as l-lactate dehydrogenase A chain, alpha-enolase, pyruvate kinase isozymes M1/M2, and malate dehydrogenase play an important role in involved energy generation and ATP synthase subunits alpha and beta play an essential role as ATP synthesis-associated proteins. The chaperone proteins such as the heat shock protein family, serpin H1, and protein disulfide isomerase A6 exhibited regulated relationships and multiple targets in phthalate chemical probe affinity experiments.

## 3. Methods and Experimental Section

### 3.1. Materials and Chemicals

Chemicals and reagents such as EDC, sodium acetate, DL-dithiothreitol (DTT), sodium cyanoborohydride (NaBH_3_CN), and trifluoroacetic acid (TFA) were purchased from Sigma-Aldrich (St. Louis, MO, USA). Acetonitrile (MeCN), ammonium hydrogen carbonate (NH_4_HCO_3_), sodium hydroxide, hydrochloric acid, sodium dodecyl sulfate (SDS), ethanol, andurea were obtained from J.T.Baker (Phillipsburg, NJ, USA). Formaldehyde solution (36.5%–38% in H_2_O), sodium chloride (NaCl), formic acid (FA), potassium chloride (KCl), sodium dihydrogen phosphate (NaH_2_PO_4_), potassium dihydrogen phosphate (KH_2_PO_4_) and iodoacetamide (IAM) were purchased from Sigma (St. Louis, MO, USA). Formaldehyde-*D*_2_ (20% solution in D_2_O) was obtained from Isotec Corp. (Miamisburg, OH, USA). Trypsin was purchased from Promega (Madison, WI, USA). Phthalic acid, APTES, potassium bromide (FTIR grade), and NHS were obtained from Alfa Aesar (Heysham, LA3 2XY, UK). Water was deionized to a resistance of 18.2 MΩ using a Millpore water system.

### 3.2. Phthalic Acid Chemical Probes Syntheses

A total of 200 mg of silicon dioxide (SiO_2_, 400 mesh, approximately 40 μm; Acros organics, Geel, Belgium) was activated once using 0.5 M HCl and 0.5 M NaOH. It was then washed with distilled water and ethanol to remove and evaporate HCl and NaOH. APTES (5% in ethanol) was silanized on the surface of SiO_2_ over 12 h, followed by washing twice with 1 mL ethanol and baking in the oven at 50 °C. Next, 10 mg phthalic acid was activated using 13 mg EDC and 5 mg NHS in 1 mL deionized water. The activated phthalic acid solution was poured and mixed with APTES-modified SiO_2_ in the same Eppendorf tube over 12 h. Finally, deionized water and ethanol were used to wash modified SiO_2_.

### 3.3. Characterization of Chemical Probes by Infrared (IR) Spectrometry

APTES-modified SiO_2_, generated phthalic acid SiO_2_, phthalic acid, and SiO_2_ were ground with KBr (FTIR grade) to generate flat wafers under pressure. These were analyzed using IR spectroscopy using a Perkin-Elmer Spectrum RX1 spectrometer.

### 3.4. NRK-52E Cell Culture and Protein Concentration

NRK-52E cells (CRL-1571; American Type Culture Collection, Manassas, VA, USA) were cultured in Dulbecco’s modified Eagle’s medium (DMEM, Sigma-Aldrich, St. Louis, MO, USA) supplemented with 1% penicillin (Gibco, Grand Island, NY, USA) and 5% fetal bovine serum (FBS). The cells were cultured in a 100-mm dish, in a 5% CO_2_ incubator at 37 °C until they reached 80% confluence. Then, they were lysed in modified RIPA buffer containing 150 mM NaCl, 50 mM Tris-HCl, 1% NP-40, 0.1% SDS, and 1 tablet/10 mL of Roche mini complete protease inhibitor cocktail at pH 7.5. Finally, the concentration of protein in the NRK-52E cell lysate was determined using the Bradford assay (Thermo, Rockford, IL, USA).

### 3.5. Conditions for NRK-52E Cell Lysate with APTES-Modified and Phthalic Acid Chemical Probes

APTES-modified chemical probes (Probe 1) and phthalic acid chemical probes (Probe 2) were weighed and 10 mg of each was added to different Eppendorf tubes, with 100 μg of NRK-52E cell lysates, whose protein concentrations were determined by the Bradford assay. A total of 400 μL of phosphate-buffer saline (PBS; 137 mM NaCl, 2.7 mM KCl, 8 mM NaH_2_PH_4_, and 1.4 mM KH_2_PO_4_) was used to dilute NRK-52E cell lysate and Probes 1 and 2, followed by incubation at 37 °C for 4 h. After removing the supernatant by centrifugation, the collected chemical probes with bound proteins were washed with 200 μL of PBS buffer and the procedure was repeated thrice.

### 3.6. Direct Tryptic Digestion and Dimethyl Labeling

After removing the supernatant by centrifugation and vacuum drying, the lyophilized powder was dissolved using 100 μL of 50 mM NH_4_HCO_3_ and 48 mg urea with 5 μL of 0.1 M DTT and incubated at 37 °C for 1 h. Subsequently, 5 μL of 50 mM IAM was added to the sample and it was maintained at 4 °C for 2 h. After reduction and alkylation, the sample was diluted to less than 2 M urea with 400 μL of 50 mM NH_4_HCO_3_, followed by digestion with 0.2 μg trypsin. After 4 h, 0.2 μg trypsin was treated once at 37 °C for 18 h. After digestion, tryptic peptides were eluted from the chemical probes using 50% and 95% MeCN and collected by drying in a vacuum centrifuge to remove NH_4_HCO_3_. Subsequently, the residue was redissolved in 180 μL of 100 mM sodium acetate at pH 5.5. The digested peptides bound to Probe 1 were labeled using 10 μL of 4% formaldehyde-*H_2_* and those bound to Probe 2 were labeled using 10 μL of 4% formaldehyde-*D_2_* with vortexing for 5 min. Further, 10 μL of 3 M sodium cyanoborohydride, which acts as a reduced reagent, was added to the tryptic peptides bound to Probe 1 and Probe 2 for 1 h. The labeled solutions were adjusted to pH 2–3 using 10% TFA/H_2_O for reverse-phase chromatography on a C18 column desalting kit produced in-house. Finally, the eluted fractions were vacuum dried for nano-LC-MS/MS.

### 3.7. Nano-LC-MS/MS Analysis and Mascot Database Search

The vacuum dried fractions were redissolved in 50 μL of 0.1% FA in H_2_O and analyzed using Thermo LTQ Orbitrap Discovery (Thermo Fisher Scientific, San Jose, CA, USA). Reverse-phase nano-LC separation was performed using a Waters ACQUITY nano flow system (nano UPLC, Waters Corp., Manchester, UK). A total of 3 μL of sample from an eluted fraction was loaded onto a C18 capillary pretrapped column (20 mm × 180 μm) and separation was performed using a Waters BEH C18 column (i.d. 75 μm × 150 mm, 1.7 μm particle size). The UPLC flow rate was set at 5 μL/min (loading pump) and 300 nL/min (gradient pump). The mobile phases were (A) 0.1% FA in water and (B) 0.1% FA in 100% MeCN with the lnear gradient followed the following sequence: from initial 5% (B) in 2 min, 5%–40% (B) in 40 min, 40%–95% (B) in 8 min, and held at 95% (B) for 2 min. The peptides were detected by applying a voltage of 1.8 kV in the positive ion mode. The survey scan mode was set at *m*/*z* 400–1600 Da in the Orbitrap (resolution = 30,000) and the selected peptides were detected in the MS mode with 5 high-intensity signals and transferred into a collision-induced dissociation (CID) chamber for MS/MS fragmentation. The b-ion and y-ion fragmentations were performed using an adjusted CID mode with a collision energy of 35 V. The MassLynx 4.1 and Global Protein Lynx softwares integrated UPLC loading and analytic pump. The Xcalibur software (version 2.0.7, Thermo-Finnigan, Inc., San Jose, CA, USA) was used to manage the mass spectrometer and for data acquisition. The acquired raw data for MS and MS/MS spectra were converted using Mascot Distiller software (version 2.4.2.0; 64 bits, Matrix Science Ltd., London, UK). The software parameters were as follows: Orbitrap_res_MS2 (default parameter setting) for peak list transformation and Rodentia (rodents) taxonomy in the Swiss-Prot database for a Mascot search engine. The Mascot search program [33] parameters were as follows: Allow up to zero missed cleavages for tryptic digestion; dimethylation [MD] quantitation, carbamidomethyl cysteine as the fixed modifications, oxidized methionine and amidated asparagine/glutamine as the variable modifications; and mass tolerance of 0.1 Dawith precursor ions and 0.8 Da for fragment ions. Peptides that had a charge of 2^+^ and 3^+^ and a Mascot ion score higher than 20 (*p* < 0.05, individual peptides) were selected. Subsequently, quantitative ratios of Probe 2/Probe 1 were used to generate a protein list.

### 3.8. Pathways and Networks Analyses of Protein-Protein Interactions

Ingenuity Pathways Analysis (IPA) [34] was used to demonstrate the pathways and networks of protein-protein interactions such as chaperones and mechanisms involved in ATP synthesis.

## 4. Conclusions

In conclusion, we demonstrated the use of phthalic acid chemical probes to investigate direct bindings and protein-protein interactions using IPA networks software. Based on D/H ratios <1, it was possible to exclude most proteins with non-specific binding by this technique. Furthermore, the raw data of proteins list based on D/H ratios showed that phthalic acid chemical probes can be used to demonstrate specific affinity binding. We will still need more evidence such as that provided by pathway network by MS using the multiple-reaction monitoring scanning mode (MRM), western blotting, co-immunoprecipitation (co-IP), and mRNA expression assays to confirm and validate the following results: TCA cycle and ATP generation-related proteins, chaperone proteins, and galectin family proteins. We believe that chemical probes can be designed using convenient synthetic procedures to generate good binding sites with the assistance of computer modeling.

## Figures and Tables

**Table 1:** Statistic classification of phthalic acid affinity proteins with significant ratio in NRK-52E cell lines.

UNIPROT accession No. a	Protein identification	No. of peptides b	Molecular mass (kDa) b	H/L(I) c	H/L(II) c	H/L(III) c	Average	Std
Score > 90 and at least the ratio (D/H) > 3 of two experiments								

P85972	Vinculin	2	119.5	9.8	5.0	4.4	6.4	3.0
P62630	Elongation factor 1-alpha 1	13	51.9	10.0	4.1	4.6	6.3	3.3
P04636	Malate dehydrogenase	3	36.9	5.3	5.9	6.0	5.7	0.4
P11980	Pyruvate kinase isozymes M1/M2	4	59.4	4.9	8.5	3.0	5.5	2.8
P07150	Annexin A1	7	40.1	7.3	3.6	4.8	5.2	1.9
P04642	l-lactate dehydrogenase A chain	3	37.6	5.1	3.3	5.5	4.7	1.2
Q6P9V9	Tubulin alpha-1B chain	11	51.4	6.1	3.5	4.3	4.6	1.3
Q63081	Protein disulfide-isomerase A6	5	49.6	7.2	3.7	2.4	4.4	2.5
P04764	Alpha-enolase	13	48.6	5.4	2.9	4.1	4.1	1.3
P29457	Serpin H1	12	47.7	5.6	3.6	3.1	4.1	1.3

Score > 80, and at least the ratio (D/H) > 3 of one experiment								

P15999	ATP synthase subunit alpha	3	60.8	17.9	1.9	1.5		
P34058	Heat shock protein HSP 90-beta	3	85.9	13.3	2.9	3.5		
P69897	Tubulin beta-5 chain	2	50.5	12.8	4.3	n.a.^d^		
P04797	Glyceraldehyde-3-phosphate dehydrogenase	3	36.9	12.0	5.8	n.a.		
P62963	Profilin-1	3	15.4	7.5	4.0	2.2		
Q5FVM4	Non-POU domain-containing octamer-binding protein	2	55.7	6.7	0.5	1.2		
P63018	Heat shock cognate 71 kDa protein	4	72.7	5.1	3.5	5.7		
P04785	Protein disulfide-isomerase	5	58.9	5.0	1.7	1.8		
P06761	78 kDa glucose-regulated protein	5	74.4	4.9	2.9	2.1		
Q66HD0	Endoplasmin	4	95.3	4.7	3.6	n.a.		
B0BN93	26S proteasome non-ATPase regulatory subunit 13	5	43.8	3.6	6.9	3.5		
P10719	ATP synthase subunit beta	6	57.0	3.2	1.1	2.1		
P50399	Rab GDP dissociation inhibitor beta	3	52.2	3.2	4.1	n.a.		

Score < 80 and at least the ratio (D/H) > 10 of one experiment								

P48500	Triosephosphate isomerase	2	28.0	n.a.	27.2	n.a.		
Q5RKI1	Eukaryotic initiation factor 4A-II	2	47.3	n.a.	14.7	n.a.		
P08699	Galectin-3	2	27.4	n.a.	12.0	n.a.		
P11762	Galectin-1	2	15.4	39.9	9.2	14.0		
Q923J6	Dynein heavy chain 12	2	366.7	27.6	n.a.	n.a.		
P63326	40S ribosomal protein S10	2	19.3	14.2	11.3	7.6		
P05964	Protein S100-A6	2	10.4	11.4	n.a.	5.8		
P16617	Phosphoglycerate kinase 1	2	46.2	10.8	6.9	5.6		
P11884	Aldehyde dehydrogenase	2	57.9	n.a.	n.a.	13.1		
P16638	ATP-citrate synthase	2	123.6	n.a.	13.9	n.a.		

a Protein accession number obtaining from UNIPROT database [40];

b No. of protein peptides and molecular mass according to the MASCOT software with Swiss-Prot database;

c Ratio value of each experiment according to the result of MASCOT distiller software;

d n.a.: not applicable.
